# Theorising Human Development in Adult Life: A Complex, Multidimensional, Dynamic, Situated Model

**DOI:** 10.1007/s12124-025-09946-z

**Published:** 2025-11-14

**Authors:** Tania Zittoun, Alex Gillespie

**Affiliations:** 1 University of Neuchâtel, Neuchâtel, Switzerland; 2 London School of Economics and Political Sciences, London, UK

**Keywords:** Sociocultural psychology, Adult development, Lifecourse, Diary, Multiresolution design

## Abstract

Although it is well established that human development is a lifecourse process, little is still known about development in the adult years. Few attempts have been made to develop integrative understandings of what people learn across various domains, such as work, hobbies and family. Also, adult life is addressed theoretically and empirically usually only on short life periods or through retrospective interviews. In this paper we propose a theoretical model that draws on a sociocultural tradition and that provides us with a series of concepts that enable us to describe and understand developmental dynamics in the course of lives of people, as these unfold in changing sociocultural environments. We put them at work on an only set of data, diaries written over more than twenty years. The article first introduces a theoretical framework for development across domains, and then puts it at work on two contrasting diaries. Altogether, the paper proposes a complex, longitudinal, multidimensional, dynamic, and situated model of human development in the lifecourse, contributing both to developmental literature and to methodological advancement.

Although it is well established that human development is a lifecourse process, little is still known about development in the adult years. Indeed, psychology has tended to study cognitive and emotional development outside of its natural context, such as in the laboratory (Baltes et al., 1999, 2006; Chang et al., 2023), or to examine learning and development in the workplace, as expertise (Montero, 2016), or in the family life, as part of the lifecycle (Erikson, 1959; Fuller-Iglesias et al., 2015). Few attempts have been made to develop integrative understandings of what people learn in these domains (i.e., work and family) and others (such as hobbies). We need to account not only for what happens in this or that life domain, but understand what happens across domains, and to encompass the wholeness of human experience, both synchronically and diachronically. So far, such issues have been addressed theoretically and empirically only on short life periods (Crafter et al., 2019; Gillespie & Zittoun, 2015; Theisen-Womersley, 2021; Zittoun et al., 2013; Zittoun & Gillespie, 2015a), or through retrospective interviews (Hviid, 2022; Pedersen, 2025). What has been missing for developing a fully-fledged theory of adult development is access to longitudinal data running over decades. Addressing this gap, we draw on a newly assembled corpus of online diaries spanning more than 20 years, providing a rare longitudinal window into adult development as it unfolds in everyday life (Bernal Marcos et al., 2024).

In this paper we propose a theoretical model that draws on a sociocultural tradition and that provides us with a series of concepts that enable us to describe and understand developmental dynamics in the course of lives of people, as these unfold in changing sociocultural environments. The article first introduces a theoretical framework for development across domains, and then puts it at work on two contrasting diaries. Altogether, the paper proposes a complex, longitudinal, multidimensional, dynamic, and situated model of human development in the lifecourse, contributing both to developmental literature and to methodological advancement.

## A Theory of Human Development in the Lifecourse

Our approach is anchored in dialogical sociocultural psychology, that assumes the mutual constitution of persons and their environment (Marková, 2016; Valsiner, 2021). This implies both that human capacities develop through interactions with others as well as with objects and discourses built by others, in specific geographical, social and symbolic settings, but also that people can resist and transform other people, their guidance, or their environment; it is thus a transactional approach (Dewey, 1946). This approach is fundamentally historical (processual or developmental) and confers a central role to semiotic dynamics: it examines how people make sense of things and events, via the internalisation of signs, which enable elaborating their experience and communicating about it to others (Cole, 2023; Valsiner, 2024).

This approach finds an metatheoretical frame in open dynamic system theory, which accounts for the fact that a person in their environment, or the many activities in which they engage in their lifespace, evolve through complex interactions, escaping linear causalities, yet according to some basic principles. According to this metatheoretical approach, people’s activities stabilise along attractors, that is, states of dynamic equilibrium, which may, in case of too strong perturbation, or rupture, become unbalanced and require new equilibriums – along new attractors, or by establishing new systems (Van Geert, 2009, 2019; Witherington, 2007, 2015). Change and development thus occur during these processes of equilibration (Piaget, 1977; Valsiner et al., 2009). Of course, one may question the linearity of such process once we account for humans, whose experience is not only biological, but also of symbolic nature, or semiotically mediated (Gillespie & Zittoun, this issue; Stenner, this issue; Valsiner, this issue).

Given the metatheoretical status of this model, we need however to define a series of concepts that account for the level of description that is human development in situated complex evolving environments. Authors engaged in such endeavours include classical psychologists such as Alfred Schütz (Schuetz, 1944, 1945; Schütz & Luckmann, 1973; Zittoun & Gillespie, 2015b) and especially Kurt Lewin (Lewin, 1936, 1940, 1951). Their work, often quoted, needs however to be updated and completed to solve some of their inconsistencies and to integrate issues of semiotic processes, an effort started by others (Valsiner, 1998) and that we continue here. Therefore, we clarify the vocabulary and the grammar of our theory of development drawing on these sources, which all share epistemological assumptions (e.g., dynamic nature of human experience, dialogicality, role of semiosis). Our goal is to propose a vocabulary to describe a persons’ many activities, their social situatedness, the material and symbolic nature, what can be learned (or lost) or developed within each or across them, and what overall transformations emerge. This comprises a series of components, and of dynamics.

### Components

People live in the world; they experience it as physical or material environment, as it is inhabited by others, and for its meanings and the affects it raises. In other words, it is experienced as material, social and symbolic places. This world is made of subregions of different scales, which have material, social, symbolic or affective (subjective) boundaries. Hence, there are nation-states, geographical places (plains, mountains, cities), institutionally regulated places (schools, hospital) or more private ones (one’s bedroom or one’s garden). These, we call places, or settings (Brown & Reavey, 2020; Cangià, 2021).

People’s relevant portion of the world at a given time is their lifespace (Lewin, 1951); their lifespace includes material and symbolic aspects, and include these currently present, but also past memories and future expectations. Even the lifespace at a given time can be further subdivided, in what Lewin called “regions” with specific boundaries. These boundaries do not need to correspond to the actual social and material ones. People experience distinct environments depending on what activities they engage in (e.g., working or relaxing at home), what social rules structure them (or not), how they feel experienced by others and how they define themselves (or what aspects of themselves they activate), which skills and knowledge they engage, and how this makes sense, how that feels to them. We call these more subjective subparts of the person’s lifespace “domains of conduct”. One can thus use the same room (a material setting) to work, to discuss with a friend, or to play online chess (three different domains of conduct). We use the term “domain” because it is generally used in the literature; we use the term “conduct” drawing on Pierre Janet where it includes not only behaviour or socially or materially mediated activities, but also psychological and symbolic processes (Janet, 2003; Zittoun, 2008).

We distinguish three types of domains of conduct, located on a continuum on how socio-culturally regulated and semiotically mediated it is, and two modalities of domains of conducts. The three types of domains of conduct are (i) *personal domains*, which are idiosyncratic (e.g., reading novels for a passionate reader, which can take place in different settings and be mediated by various books, but still generates the same quality of absorption (Benson, 1993; Nell, 1988)); (ii) *cultural domains* which involve socially shared informal knowledge systems, or cultural subsystems, related to certain social frames (e.g., having Sunday lunch with one’s family, or watching soccer with one’s group of friends (Geertz, 1972; Zittoun, 2019)); (iii) formal domains, which are cultural systems which have secondary semiotic systems, that is, a system in a different language to regulate another semiotic system (e.g., grammar to English, music notation to music) (Zittoun, 2022, 2023; Zittoun et al., 2024)^1^. All of these domains can be experienced in a *proximal* mode, that is, experienced in the here-and-now of a material and socially regulated place or setting or social frame (Goffman, 1974), or in a *distal* mode, that is via imagination (Zittoun & Gillespie, 2016).

In summary, one’s lifespace is made at any moment of a complex configuration of domains of conducts, one being usually proximal, and the other ones being distal (all the multiple worlds, experience, wishes, memories, projects relevant in the present moment). *Imagination* enables people to move across these many domains of conduct (Zittoun & Gillespie, 2016). In a state of dynamic stability, that is, involving transitive changes, the person may thus live her life in a state of relative stability. She moves easily between everyday proximal domains like work, socializing, reading, and exercising, performing proximal tasks competently while occasionally drifting into distal domains (e.g., daydreaming about past memories or future possibilities). The next section examines the conditions in which this dynamic equilibrium is challenged and generates intransitive changes, that is, development (Valsiner, 2021).

### Dynamics

When a person changes their relationship to their environment, this may generate new demands on their domains of conduct or require the creation of new ones. Hence, typically, moving to a new place - geographical mobility - often requires the transformation or expansion of domains of conduct. When moving spatial location, some domains are challenged by the new physical, material and social demands and possibilities. Immigrant women in London have to find the ingredients to cook their native meals in new neighbourhoods, or to adjust the recipe to the tastes of their new guests, transforming in turn their cooking habits – which means, their domain of conduct related to cooking has to evolve (Greco Morasso & Zittoun, 2014). A young woman, June, moving inland during the British Homefront in WWII decided to work in the fields, which required the transformation of family gardening into professional agricultural skills – that is, the transformation of an personal domain of conduct into a cultural domain, if not a formal one. June was also, for the first time, surrounded by young single men and she thus created new domains of conduct linked to dating (Zittoun & Gillespie, 2015a). However, conversely, accompanying spouses of highly mobile professionals find themselves in difficult situations in remote Swiss neighbourhoods where they cannot work (for legal reasons) and where their status is misunderstood, which prevents them from creating new social relationships – both options being easy in other international locations. Here their existing domains of conduct cannot be activated and they find themselves existentially in a “limbo” (Cangià, 2018) – in our words, in a social setting to which no personal domain of conduct corresponds. In any case, the transformation of domains of conduct – their accommodation to new settings, their transformation of modes – from informal to formal – or their challenge may also lead the person to reflect about these, thus elaborating new domains, encompassing other ones. June, for example, reflected on her new experiences and transformations: having learned farming, having had romantic relations with men, she decided to live as an independent woman at the end of the war, thus creating a new domain of conduct subsuming the two other ones (Gillespie & Zittoun, 2015; Zittoun & Gillespie, 2015a).

However, people’s changing relation to their environment can be due to other causes than geographical mobility. Important transformation of the environment can generate new demands on people’s domains of conduct: a war, the climate crisis, or legal demands (Di Donato et al., 2020). Finally, these changing relationships can be due to changes in the person – bodily or psychologically. When someone breaks a leg, their environment drastically changes and their configuration of domains of conducts are questioned. This is typically the case for older persons whose bodily strength diminishes with time; their personal domains have to be consequently reconfigured: they cannot hike anymore, for instance, and even some areas of their neighbourhood may become inaccessible (Zittoun, 2026b). Alternatively, someone’s unreachable goal to become a filmmaker can bring them to disinvest their cultural domains connected to cinema (Zittoun et al., 2024).

Eventually, all these transformations are also accompanied by the fact that certain domains of conduct, at times lived in the here-and-now of a social and material setting, can now only be reached by imagining them. They just move from being proximal domains, to distal ones (Zittoun & Gillespie, 2016). Hence an older man who cannot hike anymore can now relive his past excursions by narrating them or by viewing pictures from his hikes (Zittoun, 2026a Submitted). Some people, whose relationship to their environment is extremely stable, can nevertheless develop their domain of conducts via a variety of resources. For example, Einar, an older inhabitant of the Faroe islands, never left his small harbour town; yet through meeting sailors from all around the world, listening to radio broadcasts and reading, he expanded his distal domains of conducts, to the extent that he mastered a variety of English dialects and could identify English sailors’ home regions (Pedersen & Zittoun, 2022).

In summary, we distinguish three types of causes likely to require or catalyse change and development in people’s configuration of conduct, all questioning the dynamic balance a person has within their living environment: geographical mobility; crises or rapid change within the environment (material, social or symbolic settings); a personal rupture (due to inner or outer causes).

These induce five basic types of transformation: first, domains of conduct can appear and disappear, gain importance or lose importance. Second, a domain of conduct can become more differentiated, complex, and organised, or conversely, they can lose organisation and differentiation (Werner & Kaplan, 1963; Zittoun et al., 2024). Third, a domain of conduct can become more formal and socially regulated, as when a personal domain becomes a cultural domain and/or formal domain (e.g., gardening into agriculture), or conversely, a person’s formal domain can be lost to become everyday knowledge as in personal spheres of experience (e.g., school mathematics in everyday counting) (Zittoun et al., 2024; Zittoun & Gillespie, 2015a). Fourth, domains of conduct can fuse, split, reorganise their boundaries (e.g., formal school mathematics being used in setting up a business). Fifth, proximal domains of conduct can become distal ones, and vice-versa (Zittoun 2020).

In this article we propose to examine how these dynamics operate over decades within single life-trajectories. Our goals are threefold: (1) to demonstrate the relevance of this theoretical model; (2) to further explore the transformations of domains of conducts; and (3) to examine how people modify their own environments to reinforce these transformations.

## Methodology

To study people’s lives over the life course, we analysed naturally occurring qualitative longitudinal data (Hermanowicz, 2016): online diaries, written by people for over 20 years. As our theoretical frame assumes that development is likely to be triggered by ruptures, some of them being caused by societal crises, we defined a time span so as to include three societal crises: 9/11, the financial crises in 2008, and the COVID-19 pandemic in 2020. In addition, the span of 20 + years is likely to give access to a number of personal ruptures. Finally, because the time span was the same for all the diaries, it allowed comparing and contrasting of cases, to understand how people make sense or develop through comparable events (Pedersen, 2025; Sato et al., 2013; Zittoun, 2009).

Diaries do not give access to people’s actual experiences, but to a first elaboration of experiences, usually within a day or two. Diaries are thus almost real-time semiotic elaborations of people’s lives. Diarists take some distance from experiences, and are actively elaborating these. A diary is always a dialogical endeavour, addressed to self, other (real or imaginary readers) and more generally the world (Grossen, 2015; Lejeune, 2000; Svačinová, 2021; Zittoun, 2014; Zittoun & Gillespie, 2022). Nevertheless, because a diary is itself a process of sense-making, it reflects what domains of conduct are important to people, and what they spend their days doing or reflecting upon (Gillespie & Zittoun, 2010; Zittoun & Gillespie, 2012). If diaries are not mirroring reality, we nevertheless assume that diarists are engaged into some “authentic” writing, not fabricating lives they are not living, especially if these are written over a longer period of time (Lejeune, 1975). In that sense, then, analysing diaries gives a good enough access to people’s evolving configurations of domains of conduct over time (Zittoun et al., 2024).

We identified 420 diaries from a variety of online diary sites (such as OpenDiary, Prosebox, LiveJournal, etc.) with 11–25 years of writing between 1999 and 2024; we contacted 225 diarists who had produced good enough quality diaries, that is, who wrote regular entries for many years. Each of these persons was then personally contacted, informed about the research, and asked for consent for their diary to be analysed, with or without disclosure of the pseudonym they used for writing their diaries. Thirty-nine replied to give their consent to have their data used. We downloaded the diaries and converted them into formats suitable for mixed method analysis. After a preliminary analysis of their lifecourses (a reconstruction of the chronology of courses of life, a sketch of their current lifespaces), we selected and contacted a subset of the diarists for interviews. Eventually, we decided to focus on three diarists, two men and a woman, with contrasting experiences, and who also accepted to be met in-person. For these diarists, we kept reading the diaries up to 2025 (time of writing of this paper).

In addition, two researchers of our team, Oliver Pedersen and Maeva Perrin, undertook two fieldwork trips to visit the three diarists (in 2023 and 2024). These visits allowed for a more ethnographic approach to these diarists, who could be now observed and interviewed in their current proximal domains of conducts. Also, we could check with them our reconstruction of the chronology of their life trajectories, a necessary step in biographic analysis (Riessman, 1993; Rosenthal, 1993), and discuss our understanding of their experiences. We also asked the diarists to bring us to the settings that were most relevant to them, and we visited their houses and neighbourhoods. As a consequence, in most cases, the diarists reported in their diaries about these researcher visits; the research in that sense became a dialogical endeavour (Cornish, 2020). This combination of longitudinal qualitative data and ethnographic data, or in other words, of diachronic and synchronic data, enables us to triangulate first- and third person perspectives, in a dialogical manner; the data has thus length and depth. In addition, as we work with publicly accessible diaries, the data presented here could be potentially analysed by other researchers; here, we engaged in multiple analysis of the same dataset as part of or collaborative research practices (Cornish et al., 2007, 2013; Zittoun et al., 2007).

For this article, we focus on two diarists, as two contrasting case studies (Allport, 1942; Flyvbjerg, 2006). As we will show, these are the cases of two men of the same age who went through partly comparable life experiences, yet who made sense of their experiences in different ways, resulting in contrasting current lifespaces.

The analysis of the diaries used a multi-resolution research design (Gillespie et al., 2024). This is a mixed method design with a single dataset. Specifically, the same diary texts were analysed both quantitatively and qualitatively, with recursive movement between both analyses. The quantitative analysis was used to ‘zoom-out’ and view patterns across the entire diaries. The qualitative analysis was used to ‘zoom-in’ and analyse specific episodes in the diaries. The macro quantitative patterns guided us to episodes for qualitative analysis; and the micro qualitative analysis suggested abductive insights to explore in the macro patterns.

The computational analysis was done using Python. Specifically, we used established algorithms for measuring: number of words, sentiment, vocabulary size, and semantic complexity. Topics were identified based first on a qualitative reading, identifying key words used by the diarist, and then creating word lists pertaining to the targeted topics.

The two cases are partly anonymised only: Ernest is a pseudo invented by us for one of the diarists, and we deleted or hid personal information of his case. Brickpaver is the pseudonym the other diarist uses for his writing, which we maintain according to his own will.

In what follows, we present the case analytically, following the theoretical frame proposed above. What we wish to demonstrate is two contrasting case studies: how two men, with relatively comparable trajectories in socio-demographic terms, construct over time very different lifespaces through their different sensemaking within ruptures. Through this, we wish to demonstrate the dynamics of how people’s configurations of domains of conduct evolve with time, and the processes through which they maintain an overall equilibrium through their transactions with their environment. Thus, analysis reveals a general principle of development in the course of adult lives. In order to do support our demonstration, we draw primarily on our analysis of the diaries, which we complement with data from the in-situ ethnographic work.

## Comparative Study of Two Contrasting Cases

Ernest was born in 1951 in a southern state of the USA; the oldest of a family of three, he studied journalism; he worked as journalist, as educator for people with special needs, as teacher, and as librarian until his retirement. He never married, and after some losses in his family he moved back to his mother’s house to take care of her until her death. Alongside these activities, he developed an interest in photography, first linked to his job as journalist, then more related to a growing fascination for the surrounding nature and especially flora. He now lives in a resort for older retired persons, yet is quite isolated from other residents.

Brickpaver was born in 1952 in the state of New York, also with two siblings. He studied watchmaking, and subsequently entered in a car factory where he worked most of his life until an early retirement in his mid-50s, by then having moved to a more southern state. He lived for seven years with a partner who died of AIDS in the 80s. Next to his work, he developed a series of interests in rowing, homemaking, gardening and collecting bricks, fire hydrants and music boxes. He lives alone with cats in a lively neighbourhood where he has an intense social life.

Ernest and Brickpaver share many similarities: they are both men born in the early 1950, who have been diarists for more than 25 years, which supported them to become very reflexive about their experiences; they are both retired, have never been married and have no children, and live alone, devoting much time to their longstanding interests. They also both experienced the major sociopolitical crises of their adult lives – 9/11, the economic recession of 2008, and the COVID 19 pandemic in the early 2020s. Additionally, they are both sensitive to political transformations and climate change. Also, each of them has been affected by personal ruptures, including the loss of partners and parents, and professional ruptures.

However, Ernest and Brickpaver have also very contrasting experiences. Although both write about their present and their past, Brickpaver appears more open to new experiences and challenges. Ernest lives with minimal social interactions, while Brickpaver has an intense social life. Ernest’s love for photography of nature brings him to have a meditative outdoor life, while Brickpaver is more mechanical, and often building things. Let us try to account for these contrasting lifespaces.

### Understanding the Transactions with the World

It is likely that the main source of people’s development, or at least, what triggers or catalyses changes, are modifications of the equilibrium of their configurations of domain of conduct, due to changing transactions with the environment. These may be primarily due to crises and ruptures, or to mobility.

#### Crises, Ruptures and Domains of Conducts

A first look at the visualisations of their dairies of the two men shows that different domains of conducts have taken changing relative importance over the years, these relative importances being quite clearly linked to specific crises or ruptures, which we treat here together.

**Fig. 1:**
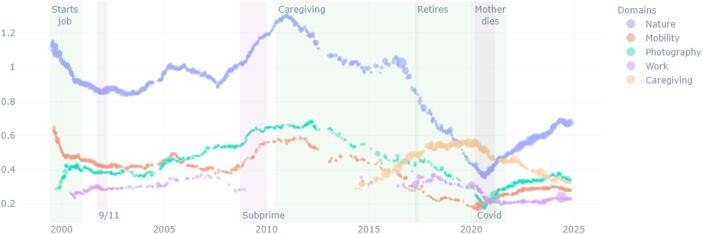
Ernest, percentage of words per domain over time

**Fig. 2:**
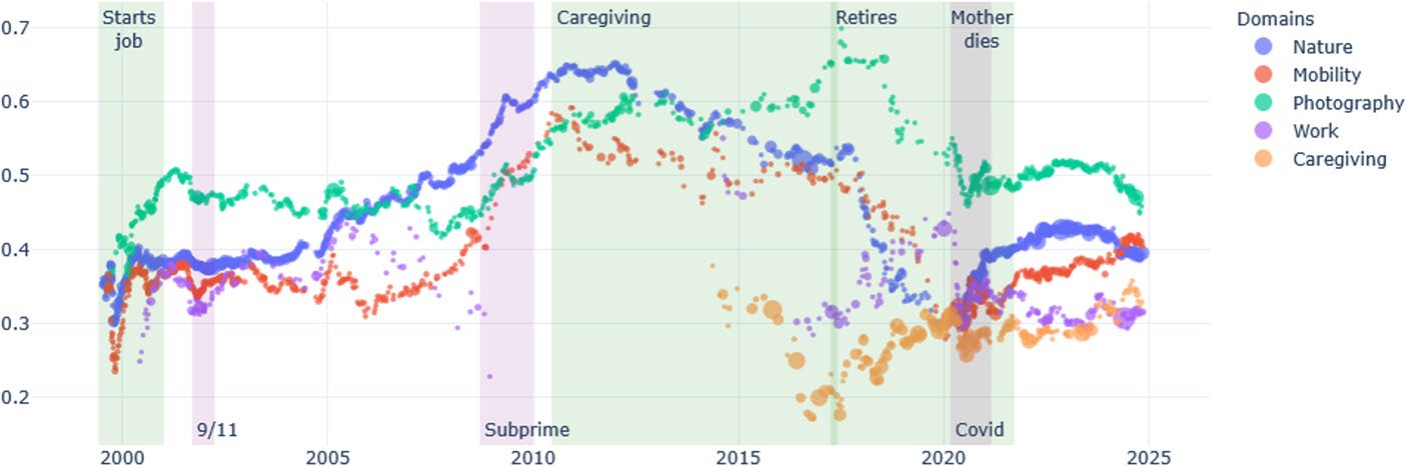
Ernest, sentiment positive/negative per domain over time

Over the last 25 years, the main ruptures in Ernest’s life are due to changes in is personal immediate environment: the illness of his mother and her death, and his retirement. Ernest’s interest in photography and in nature takes great importance in his diary, and the space it occupied in the diary (the percentage of words) progressively increased as his professional life as librarian is stabilised. Photographing together with his interest for nature lose importance as he has to take care of his ill mother, and after she dies, they take more importance again (see Fig. 1). In terms of relative subjective importance, identified by the overall “sentiment” of these domains, it appears that his most positively valued themes vary over time: nature as is it taken care for, flora, or photography are alternatively more positive; the domain of caregiving is expressed most negatively (Fig. 2).

**Fig. 3:**
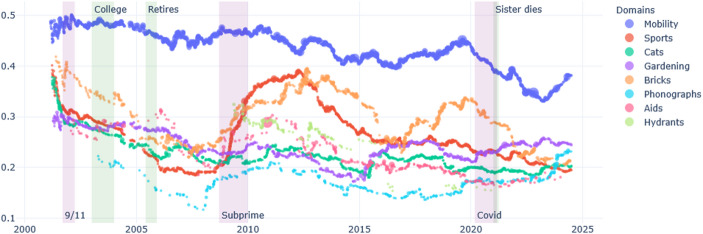
Brickpaver, percentage of words per domain over time

**Fig. 4:**
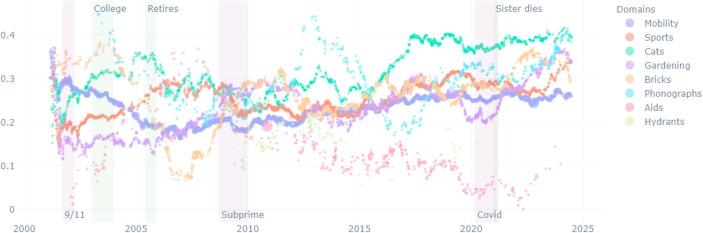
Brickpaver, sentiment positive/negative per domain over time

Brickpaver’s life is marked by crises that occurred before he started writing the diary: his own coming out as a gay man in the early 80s, the AIDS crises in the 90s, and the problems that affected the car industry in which he worked. His retirement in the early 2000s was a long- planned dream. He was also affected by a tornado in 2011, when he supported his community with voluntary work. Brickpaver is a man of many interests. He constantly writes about his projects, his travels, gardening, and his reflection on AIDS. After his retirement, various domains of conducts take more importance: first his brick collection and sports (rowing mainly) for about ten years, and progressively, his interest fin gardening increases (Fig. 3). However, when we examine the emotional tone of these domains of conducts, we see that in the past twenty years most domains took a positive tone, especially a new interest for cats, while his reflection on AIDS becomes increasingly negative (Fig. 4).

Hence, in these two men’s lives, it seems at first glance that they both have been marked by events experienced as ruptures, some being caused by societal crises (e.g., AIDS and COVID, but also, for Brickpaver, economic recession hitting the car industry, and for both, climate change). Both have also experienced more personal ruptures as the death of a parent, or a professional change. Hence, societal crises and personal ruptures play and important role in the unfolding of the lifecourse; they require new equilibrium between oneself and the environment, and are occasions for change and development. However, the consequences of the ruptures are not linear; they affect some domains more than others, and so they may also have unpredictable outcomes.

#### Geographical Mobility, Psychological Change

Mobility plays an important role in psychological transformation (Gillespie & Zittoun, 2015; Märtsin, 2019). Both Ernest and Brickpaver have experiences of geographical movement (see “mobility” in Figs. 1, 2, 3 and 4), and, in both cases, these have been important for their development.

Ernest, who at first sight seems least mobile (he remained in the southern part of the USA all his life) has experienced many inner mobilities. He summarises them in one entry from 2022:

I remember how exciting it was when we moved to our new house in the suburbs of New Orleans in 1961. It was a brand-new, brick house with big bay windows and a small front porch, nestled among live oak trees. What a thrill for a ten-year-old to witness the movers unloading our furniture and boxes into this grand new “home”. […]

Moving days after that were much less formal affairs. In the years after college, I lived first in an old and timeless boarding house in Columbia, SC, in a upstairs room filled with antique furniture. […] My next big move involved renting a Ryder truck, and, with the help of my best friends, loading up and heading for North Carolina. […] I returned to South Carolina just eight months later […]. What followed during the 1980s was a long odyssey of graduate school, teaching, traveling the country and wandering in and out of employment as I seemed doggedly determined, or fated, to remain without a stable job or anchorage for the rest of my life, without that place to truly call “home.” I didn’t find it until 1995, in my mid-forties. […]

Tonight, as I again think about that huge moving van, […] I can say for certain that whatever form my single, solitary moves took, and whatever the goal or lack of one, they were equally momentous and life-changing events. […] Moving day, no matter what, always signified hope, a new dawn and starting over.

A few years later, he moved to a house that he thought would be his final home, and where he eventually just stayed a couple of years, until moving in with his mother; after she passed away, he sold it and moved to his current flat. Hence, Ernest’s mobility seems both chosen, as way to develop his professional career, and reflecting his professional and perhaps relational variations. They are as much a trigger his changes, as corresponding to what seems to be an inner state of opening to transformation – hope and the possibility of a new beginnings (Bernal Marcos et al., 2025).

Brickpaver has a different experience of mobility, with an early life-changing motorbike trip to Europe. As he will later comment, “That summer set the course of my life. I grew up with the old saying ‘He who travels fastest travels alone’. Unconsciously I set my sails to travel through life as a solo voyager”. This, in some ways, opened up a general principle of mobility. This was translated by a variety of moves. Trained as watchmaker in the early 1970s, Brickpaver eventually started working for GM in 1972 under precarious conditions. He moved in the 1990s to live with his partner, and after his death, to Tuscaloosa, Alabama, for work and where he bought a house. Due to professional requirements, he was transferred to a plant in Bowling Green where he lived for 13 months, before moving back to his house in Tuscaloose to retire. Nevertheless, throughout his life, he was endlessly traveling on weekends and holidays to pursue his various domains of conduct; conventions of collectors, or private owners of bricks or fire hydrants. Here is a quote from a typical entry where he writes about his trips in September 2004:

The one escape I look forward to every year is the trip I make to Pennsylvania for Summer Camp. Basically this is a group of guys who meet for 5 days to let loose at a rural kids summer camp. […] It is sprinkling as I leave Bowling Green. This will be the pattern for the rest of the day. I will just be ahead of this storm, driving under heavy, leaden gray skies. There is no easy way to get to camp from Bowling Green. I head up RT 65 to the Blue Grass Parkway. I take this road to Lexington and then a bunch of other local roads to by-pass the city. Finally I get back on the interstate system at RT 64. […] It is night driving through the mountains that I hate. Also I’ve lost an hour changing back to Eastern Time. It is just outside of Morgantown I stop to call it a night. I find the perfect Microtel, it is just across the street from an Outback! They are full, and so is the Econolodge next door. My best bet is to backtrack five miles to the Comfort Inn. There is a line of people waiting to check in. It looks like we are all in the same boat. I almost gasp when I’m told the best rate I can get is $74.00 for the night. I’m so sick of driving. Do I need to risk having an accident just to save a few bucks? NO!! There is a restaurant next door that stops serving at 9:00. It is 8:45 as I walk in the door. I go to the bar and order a nice cold draft Budweiser, along with a burger and fries. Now I can relax!

Brickpaver’s depictions are almost in real time, distal memories become lived proximal domains – roads, state boundaries and motels. However, these also have resonances at a more psychological level (Pedersen et al., submitted paper). Indeed, through daily writing, Brickpaver is vicariously reliving experiences, narrated in the present tense. There is thus a double-mobility: first, the original experience of geographical mobility in a proximal domain of conduct; and second, the vivid re-living in imagination, that is, as distal domain.

Hence, geographical movement requires new transactions between the person and their environment and engages psychological change (Gillespie & Zittoun, 2015).

### Mapping Out Domains of Conducts and Their Evolution

Domains of conduct evolve with time, very often as response to changes in people’s transactions with their environment. We distinguish fiver types of evolutions: they may gain or lose importance, appear or disappear; they may gain or lose differentiation; from there, they may become more or less formal; they can absorb each other or split; and they can move from proximal to distal and back.

#### Domains of Conduct Gain and Loose Importance, Appear or Disappear

First, domains of conduct can gain or lose importance, and appear or disappear. As seen in the case of Ernest, the domain of conduct of caregiving is very specific to the period of his life where he moved in with his ill mother and took care of her until she died. During this period, all the other domains of conduct lost relative space in the diary; the caregiving attracted most attention in the writing. However, this changed again after his mother’s death, when nature and photography take importance again (Figs. 1 and 2).

**Fig. 5:**
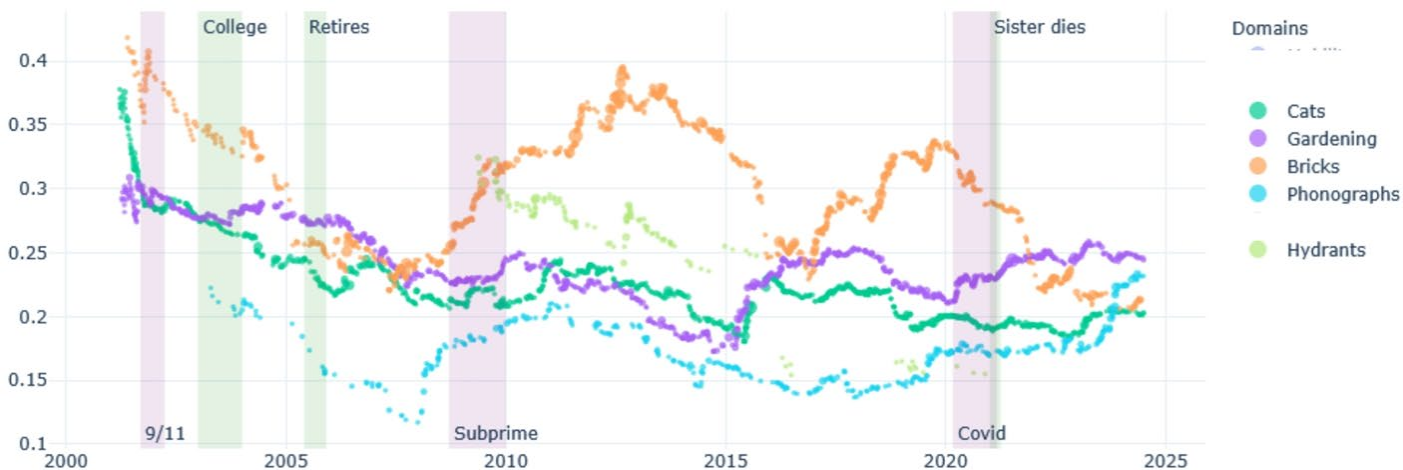
Brickpaver - Changing presence of domains of conduct

**Fig. 6:**
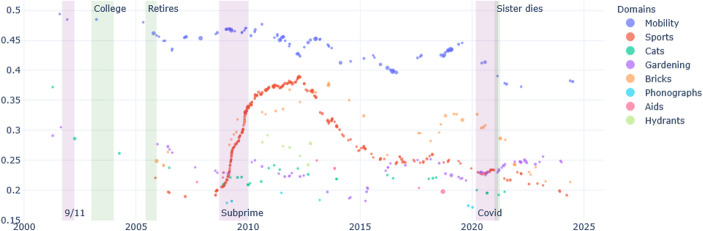
Brickpaver, percent of text, occurrences of “rowing”

In the case of Brickpaver, his collection of bricks was of great importance in the entries in the 2010s, but this domain of conduct progressively lost importance, and has recently been replaced by more space given to his collection of phonographs. This is partly due to the fact that the brick collection was a pretext to drive and meet people around the country. During the COVID, Brickpaver stopped traveling, and cancelled some meetings with other collectors (Fig. 5). Interestingly, the domain of conduct of sports, including training at the gym and rowing, made an appearance around 2010, a few years after he retired, when Brickpaver became concerned about taking on weight (Fig. 6). Hence, cessation in one domain (work) can create an opportunity for new domains to grow (sports).

#### Domain of Conduct, Differentiated and Dedifferentiated

Over time, people’s domains of conduct become more differentiated and organised; this is the classical theoretical hypothesis that can be drawn from constructivist-organismic approaches (Lightfoot et al., 2004; Werner & Kaplan, 1963; Witherington & Boom, 2019). Based on a Vygotskian hypothesis – that more differentiated thought may be manifested by more differentiated words (Vygotsky, 1934) - we operationalise differentiation and organisation using a standard measure of sentence complexity. However, the hypothesis of a linear progression would fail to account for the complexity and situatedness of life courses. Here, we argue that domains can become more or less complex, and that the overall configurations of domains of conducts can evolve in syntony, or in more differentiated way.

A first case is thus when, with time, a person sees all her domains of conducts become more complex and differentiated. This would support the idea of central competences maturing. In the case of Brickpaver, the progression seems to be linear, in all domains of conducts.

There are some variations: for instance, in domains about which Brickpaver reflects a lot, such as the AIDS pandemic, we see more change over time. Around 2016, Brickpaver goes through an intense reflection on what happened to the LGBT communities earlier. Here for instance a quote from February 2016:

Seattle Drag Show. […] The entrance was of the back alley. It was an old style gay bar with a stage/dance floor set up. […] I’m no stranger to drag shows, but this was bizarre by even my standards. This woman was dressed as a fairy princess with cut out wings, a long taffata dress held up by an ammo belt, rolling all over on roller skates. I held off getting a beer. I know how these shows go. I was correct in it started off a half an hour late. The benefit for this was a halfway house for transwomen. I learned over the course f the show the woman on roller skates was in charge of this house. The master of ceremonies goes by the name of, “Your Gay uncle” or something like that. […] As the tables and chairs were being cleared away the disco song “Gloria” blasted out. The kids ran to the floor and started gyrating. This is where the introspection kicked in. This song was at the top of the charts when I was just coming out of the closet back in the early 1980’s, probably well before most of the kids on this dance floor were born. How many nights did I shake my hips to that disco beat on the dance floor, under the strobe lights at City Lights, the gay dance club in Buffalo? There would be a group of gay “leather boys” with fantastic fans, and accompianant instruments performing on the edge of the floor. […] AIDS was just about to shatter this carefree world. Most of the people who I’m remembering from those early days are dead.

Similarly, in the following months, various events remind him of this period or trigger the exploration of this past distal domain of conduct – as when starting spring cleaning leads him to remember his house move in the 90s and soon after the death of his partner “and scores of friends to the AIDS plague”. He continues: “I’ve read where psychologists are now saying how those who have survived the plague can be afflicted with a form of PTSD. There was such a loss suffered with virtually no support from the government or society in those early days” (March 2016).

Hence, in the life of Brickpaver, his coming out as a gay man, and later experiencing the beginning of AIDS started as very rough personal domains of conduct; they became differentiated with time, including social networks, devoted clubs, clothing codes, music, dance moves, and progressively, knowledge about AIDS and analysis of the underpinning of the politics linked to it. This would suggest that complexity may be domain specific, and that indeed, it is by socialisation and mastery of specific domains that expertise deepens.

The hypothesis of domain-specific complexity is supported by our previous study on adult development (Zittoun et al., 2024). There, we have shown that sentence complexity can also vary within one domain. In the case of Ken, for instance, a progressive dedifferentiation in the domain of film watching was accompanied by a growing differentiation in the domain of international politics (Zittoun et al., 2024); this could be linked to the fact that Ken was unable to fulfil his dream to work in the film industry. Hence, a changing transaction with the world can bring to a progressive disinvestment in one domain, accompanied by the emergence of a more relevant one.

Yet even the hypothesis of a progressive complexification may not always hold. In the case of Ernest, it seems that the complexity diminishes in challenging periods across all his domains of conduct; there is long decline, and a reversing of that direction soon after his mother’s death, when he is freed from intense caring for her.

**Fig. 7:**
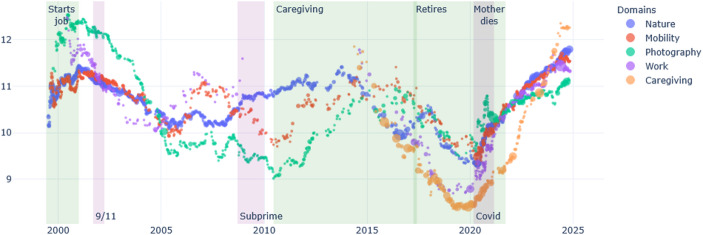
Ernest, sentence complexity per domain

Potentially the double rupture of retirement and caregiving for his mother during the COVID pandemic put much strain on Ernest, and put him under a difficult pressure (Bernal Marcos et al., 2025). During this lonely time, he found very little resources to support his usual significant domain of conducts. Interestingly, while during his mother’s illness, the percentage of words about caring augments (Fig. 1), yet the sentence complexity for discourse about caring falls (Fig. 7); there is thus a decline in complexity for what Ernest he is writing about most (caring) - which definitely points to a global decline in complexity.

It may be the case that this double rupture led him into a mild depression; Ernest indeed recurrently writes about his depression, as here in 2017:

It seems an eternity now since I was in the working world. Retirement still seems surreal to me. […] Five months after I carted off the last box of my cubicle belongings and memorabilia, […] no more…normal working life where you feel useful and are fulfilled in your work. Gone. I now wake up most mornings feeling depressed and wondering when I will free from the anguish and tribulations of seeing a loved deal with dementia.

He also mentions that he reads books about depression. This may thus be connected to the change in his writing. Literature indeed suggests that depression may be correlated with loss of specificity in autobiographical memory, which then to be expressed in more general terms (Lemogne et al., 2006; Van Vreeswijk & De Wilde, 2004).

However, an understanding of the overall configuration of experience makes us propose an alternative hypothesis. Indeed, in the case of Ernest, this trajectory goes hand in hand with the percent of his diary occupied by each domain (Fig. 1). One may propose that certain domains are more or less present or foregrounded at certain moments in Ernest’s life. Here, for instance, contrasting sentences about nature; one during the period with a higher complexity (May 2004):

This is the first day so far this year in Charleston that it’s felt like summer. I noticed that “hot heat” all of a sudden.” It’s as if you walk outside and it’s instantly summer. The air **sort of simmers**,** the warmth is suddenly all around you**, nudging close. But it feels good because it’s been so long. Since last October. **The scent of** Confederate jasmine is extra heavy in the air. You smell the damp earth, still moist from heavy rains this past weekend. Everywhere **the** ligustrum **are in bloom**. I stop to smell the sweet, but fresh and slightly **citrusy white flowers**, clustered in tiny bunches at the ends of branches on shrubs everywhere on my walks downtown at lunch. This becomes a ritual each year at this tme. Ligustrum takes me back to my childhood whenever I pass the flowers and inhale their fragrance. During the period in my life when I was 6–10 years old, we lived in a woodframe duplex apartment in a pleasantly named apartment neighborhood called **Azalea Gardens** (…).

This is contrasting with another sentence written by Ernest in February 2020, just after his mother’s death:

As much as I’ve come to dearly love the **parks and gardens** here that I frequently visit, and which were saving sanctuaries for me during the time I was caring for Mom, I feel the need to begin the rest of my life some place else.

There is a change in the richness and differentiation of the vocabulary to talk about nature, and in the second case, it becomes much more general. But this change in richness of the vocabulary is probably mainly due to the fact that, simply, Ernest is thinking about something else – his grief, his late mother, and not the beauty of nature. Soon after the richness of the depictions comes back, whether they are depicted in the present, or in the past, as Ernest remembers the beautiful places he visited as a young man. Thus, it seems that some domains have gained more complexity than others in recent years; in Fig. 7, for instance, one domain that sees sentence complexity – which indicates more complex arguments – increasing - is nature preservation. Indeed, Ernest becomes very aware of the consequences of climate change (see also Perrin & Pedersen, this issue).

Hence, the degree of differentiation of certain domains at specific points in time may depend on the overall weight of this domain in a current configuration of domains of conducts. Dealing with the death of someone, with its heavy emotional load and the psychic work it requires, becomes an attractor for the stream of thinking/writing, which then mainly turns around it. In this period, a proximal personal domain is the main attractor; the other domains are simply not explored.

#### Domains of Conduct Can Move from Personal Spheres of Experience to Formalised Knowledge

One implication of the previous paragraph is that with progressive differentiation or dedifferentiation, domains of conducts can become progressively formalised. Ernest with time turns his love of nature into a consistent formal domain about species of flowers and trees (but interestingly, his interest in photographing does not lead to an interest in the technicalities of the craft – his pictures are made with his smartphone). In contrast, Brickpaver elaborates his personal domain of being a gay man to the mastery of a complex cultural domain; and he turns his love for bricks into a systematic formal domain of knowledge about typologies, qualities and provenances of bricks.

For instance in June 2013, when Brickpaver is completing his garden paving project, he writes:

The weekend was spent working on the patio with the new bricks. I was able to get some 30 extra fancy bricks, along with the “square design” bricks my last run to Knoxville. There was not enough to fill out the two steps with that design. However, I was able to work out a design using the other brick I have plenty of. I decided to use the yellow brick for the bottom pad. That way it can be like the “Yellow Brick Road!” That was my morning Sunday; measuring and sawing bricks to lay in the herringbone pattern. I was in luck that the dimensions worked out so there was little waste. I want to research this brick a bit more. I don’t think they are Ohio brick. The guy I got them from thinks they were made by Southern Clay in Tennessee. They are not fired as hard as the Ohio Brick sidewalk pavers.

Here we see that Brickpaver not only distinguishes bricks by colour, but he knows their provenance, their composition, their mode of confection and the patterns that can be constituted with them. When he is missing information about these bricks, he engages in “research”. This is thus a domain of conduct slowly becoming formalised, both theoretically and practically.

#### Domains of Conduct May Fuse, Divide, Be Subsumed

Third, domains of conduct are usually distinct because they may be born from people’s interactions within different social settings and/or institutions, or through interactions with certain specific others. Thus, people often differentiate the way they do mathematics at school or at home, or in playing or learning situations, because the social framing renders the activities different (Schubauer-Leoni et al., 1992). However, with time, and with a change of the relative weight and pressure of different settings or social interactions, these domains can get reorganised.

In the case of Ernest, the domain of conduct of nature watching divides in time in depiction of flora, notes about animals, and concerns for the state of nature due to the climate crisis. This can indeed be deduced from the slightly distinct curves of these different domains in Figs. 1 and 2. Conversely, photographing fused with his interest to nature. He started photographing as part of his studies in journalism, after a BA in English:

I had recently graduated from college and moved to South Carolina to get started on a career in journalism, which I had my sights firmly fixed on. I was taking journalism courses at the University of South Carolina […] One of my courses at USC was Photojournalism, and in the class we learned about processing black and while film and printing our own photos for the class in a large darkroom. It was all intoxicatingly novel and exciting for me, and I loved it. (Ernest 27.07.2024)

**Fig. 8:**
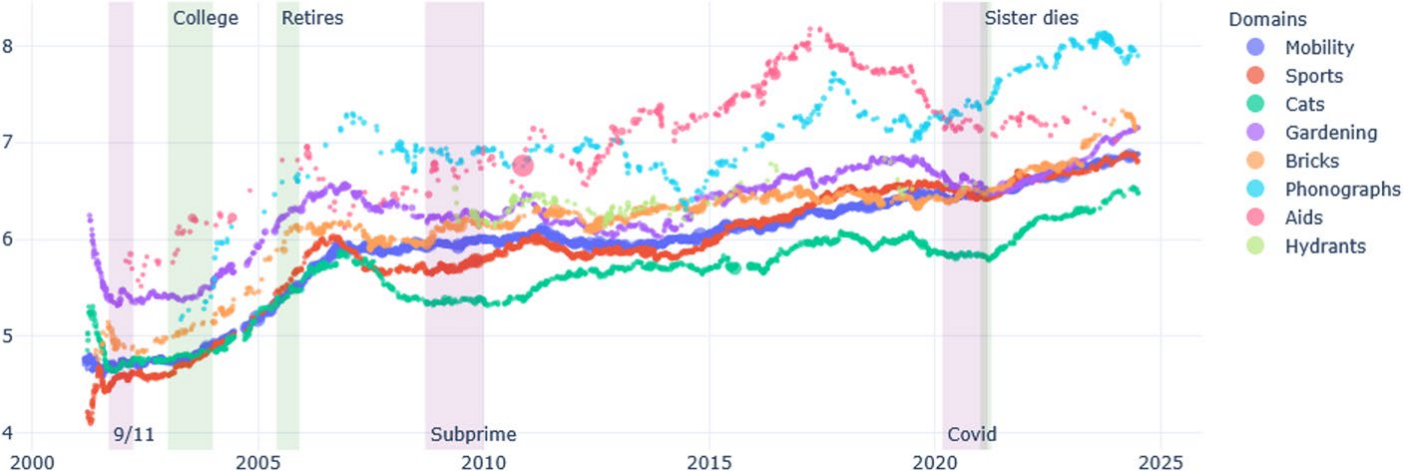
Brickpaver, sentence complexity per domain of conduct

**Fig. 9:**
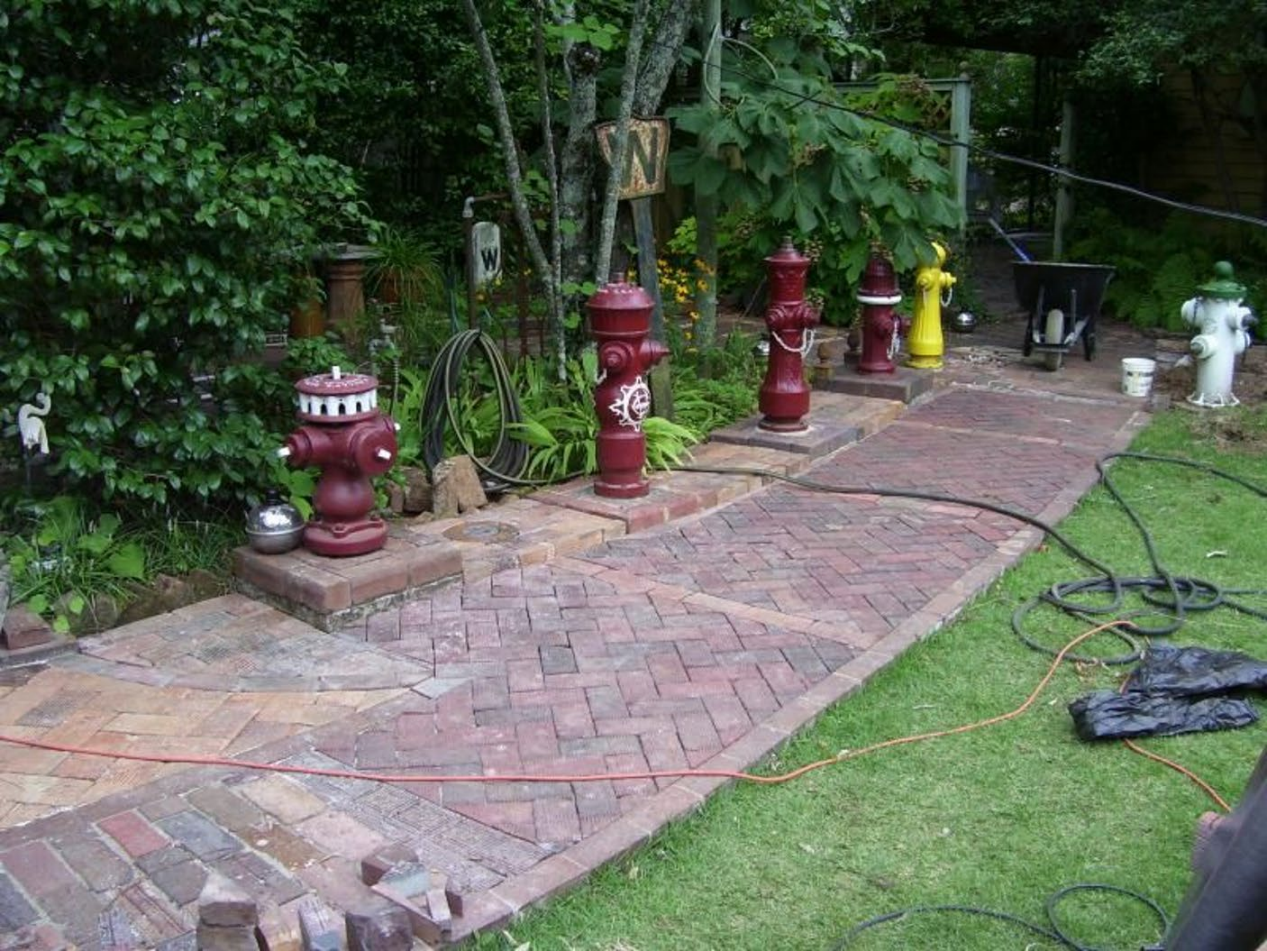
Photo by Brickpaver: brick pathway and fire hydrants, 2013

At that time, with his friends, they entered a deserted building, and he made black and white pictures of them. However, photojournalism was part of a domain of conduct related to studies, and then to his job as journalist. As mentioned, this was soon terminated. However, Ernest continued photographing and writing about it. In parallel, he often mentions the beauty of nature, of changing a light, etc. What Figs. 1, 2, 8 and 9 suggest is that Ernest’s interest in nature, and photography have relatively independent evolutions for most of his life; only since his mother’s death, do they start to evolve together – same shape of progressive positive feeling, superposed progressive complexification of the domain. And indeed, with time, Ernest shifts from taking photos of buildings and human-made structures to mainly takes pictures of nature (flowers, trees, water), sometimes next to an abandoned house. Hence, one may suggest that domains of conduct that were previously separated (photographing as part of journalism and an interest in nature) became fused.

Brickpaver has developed his various collections (of bricks, phonographs, fire-hydrants, music boxes) over a long period of time, as relatively distinct domains of conduct, each with different communities. However, he at times uses the term “project” to designate his various gardening, hydrant and brick collecting activities (see Fig. 9). Hence when a project includes creating a specific portion of garden with bricks and fire hydrants, one may say that these domains of conducts join together to become part of one new “creation” domain of conduct.

#### Domains of Conduct May Move from Proximal to Distal and Back

Fourth, people find another way to adjust to changing transactions with the environment, which is the transformation of proximal domains of conduct into a distal one, or back.

When Ernest writes about nature, he explores former personal domains of conduct, now distant one – trees and flowers of older houses or landscapes he used to explore as a child. See for instance how in October 2024 he recalls his apartment after his studies in the late 1970s:

But what made this place magical was the particular 2-bedroom unit I rented, which just happened to be in the perfect location at the far end of the complex, bordered on the front and side by a quiet patch of woodland filled with hickory and oak trees. It was totally quiet there. On summer nights the woods came alive with the mesmerizing sounds of frogs, crickets, and other insects, rhythmically droning and reaching high and low crescendos of sound. I had never heard any Nature sounds as rich and melodious.

Here, nature is explored again, albeit in a distal domain – yet with the many details and differentiation that Ernest probably only recently acquired (i.e., as an older adult).

Also, distal domains can take different roles. For instance, when Ernest has to take care of his mother, he is aware that his proximal domains are shrinking; he literally expands his lifespace by looking at the sky, via his photography:

As my world as become more circumscribed the older I get and the more I am involved in caregiving responsibilities, I treasure especially now the [brief] walks at sunset to Colonial Lake in Charleston which afford a rare break in the cityscape to reveal the splendor of infinite varieties of clouds and sunsets that are always capable of dazzling my sometimes numbed senses. When seeing those magnificent skies and clouds above the Ashley River in the near distance, I am transported away from cares and concerns momentarily. In the realm of the magnificent, the tawdry concerns and interests that obscure and dull people’s senses and appreciation of beauty, disappear into the ether and present glimpses of what worlds beyond our present knowing might be like. And this gift is offered nightly when there are clouds in the sky, not as often in the clear blue days of winter, but still frequent enough in our subtropical coastal climate. (January 5th 2011, followed by a series of pictures of clouds reflecting in the water at dusk)

Brickpaver also often revisits past experiences in the light of current ones; so he keeps turning proximal domains of conduct into distal ones. For instance, during the COVID 19 pandemic he was actively drawing on his experiences of the AIDS pandemic, which then, through resonance, became a resource to decide how to handle the current health crisis (Pedersen et al., submitted paper). Conversely, as he lives in a difficult time in 2018, Brickpaver decides to return to a brickpaving project which he accomplished in 2013 as a resource to find some calm; the imagined project, a distal experience, thus becomes a new proximal domain of conduct.

Bricks are healing so I finally got to work on reconstructing the lower part of my driveway. When the new sidewalks were installed last spring, there was not a good transition between the sidewalk and the driveway. The bricks had settled in to the point water would collect and puddle in heavy rain. […] I have acquired a number of better bricks to display in this area over what I had available when I did the installation in 2012. I think of switching out my bricks to be like buying new furniture! The start of the reconstruction project.

Hence, this section highlights the many dynamics by which domains of conduct evolve, either alone, in contrast, or in synchrony, in a given configuration of domains of conduct. The four processes of differentiation or dedifferentiation, fusion or division, appearance or disappearance, and the movement between proximal and distal, appear to be the core processes by which the overall equilibrium of a life-space evolves: they seem to be at the heart of human development.

## New Understanding of the Mutual Constitution of Self and the World

If people’s configuration of domains of conduct may be extremely diverse, and referring to places and experiences distributed in time and space, as well as exposed to pressures and demands of changing environments, how do people keep a personal sense of continuity and integrity (Erikson, 1959)? For one part, such continuity and integrity can be achieved by dynamics of sense-making and imagining, via the use of symbolic resources or by diary writing (Muller Mirza, 2026; Svačinová, 2021; Zittoun, 2006, 2014; Zittoun & Gillespie, 2022). Alternatively, continuity and integrity can be done via the use of objects and one’s material setting (Zittoun et al., 2021). This calls for attention to the dynamics of homeostasis: how can one’s diverse experiences in diverse domains can be “contained” in a whole?

This process could be a generalization or continuation of the construction of psychological unity. Developmental psychology shows us that young infants, whose physical movements and psychological dynamics are not yet regulated from within, thus bringing the child to quick states of disorganisation and anxiety, need to be contained by caring activities, such as handling and holding (Winnicott, 1991, 1994), or basic attunement (Stern, 2004; Trevarthen, 2012). This has been theorised as the construction of a series of mutually containing psychic “envelopes”, or boundaries, where family care, and symbolic systems, can support from the outside more inner, psychic or physical envelopes (Anzieu, 2003, 2007; Kaës, 2007). Along one’s life, there is a form of dynamic equilibrium between these; when one’s inner envelopes are more challenged (e.g., one experiences a ruptures) then outer envelopes, such as family and friends, can reinforce one’ s sense of continuity and integrity. In older age, when these inner envelopes become more fragile (when the body shows weaknesses, when one may have memory or cognitive losses), then close interpersonal and institutional envelopes can become more important again (Bonnet & Louchard Chardon, 2024). For instance, older people may feel more comfortable attending a daycare centre, or more supported if they enter an institution (Bonnet & Louchard Chardon, 2024; Gfeller & Grossen, 2025; Zittoun, 2026b). In other words, their configuration of domains of conduct may find a consistency from the outside, by the materially, socially and symbolically containing function of the environment.

This invites us to examine how our diarists develop external containing envelopes to reinforce the dynamic equilibrium of their configurations of domains of conduct in times of crises and as they are ageing. Both diarists are now in their mid-seventies, and both are living in the USA that goes through a series of important crises: COVID 19, the climate crisis, as well as the distressing government policies.

### Configurations of Domains of Conduct May Be Stabilised in The Material Setting

Brickpaver is a collector of bricks, phonographs and fire hydrants; he also likes to take care of his garden. What we did not anticipate from his dairy, is how much these objects constitute his homemaking. His house is fully decorated with the classical phonographs, historical lamps and associated furniture. His garden is decorated by an alley of fire hydrants, he has used his bricks to create a pathway and a wall in his garden. In addition, Brickpaver has an intense social life in his neighbourhood. Brickpaver was always a bit of an outsider. Yet in his older age, he has constructed a real home whereby his passions literally materialise the boundary of his world, yet which is also nourished by an active social network.

In other words, one modality of stabilising a complex, dynamic configuration of domains of conduct, is by creating a proximal material and social envelope that supports and reinforces it.

### Configuration of Domains of Conduct May Be Symbolically Stabilised

Ernest has less invested is homemaking. Having moved many times, he has accumulated boxes and objects, that also provide a stabilizing envelope. More interesting, however, is his relationship to nature. From his writing, we know how much Ernest is attentive to describing trees, lights and atmospheres; he also often likens them to poems or songs. Yet as our research team went to visit one of his preferred parks with him, Ernest also showed them how exactly they should frame a photograph, to create the perfect balance of trees and water reflections. Also, these pictures he takes are often the same. Our impression is that Ernest is not so much appreciating the nature as it is, as turning it into his own creation, or recreating a poetised version of it. In other words, one could say that Ernest is reinforcing his configuration of domains of conduct by this symbolisation - creating distal, symbolic envelopes that confers stability to his equilibrium.

Overall, these longitudinal analyses reveal that, over time, not only do people’s changing transaction with their environments change themselves, but also how people can transform their environments so as to stabilise their lifespaces and thus themselves.

## Discussion and Conclusion

This article contributes to an integrative sociocultural psychology of the lifecourse, especially highlighting developmental dynamics taking place within the evolving transactions people have with their changing environments. Drawing on an open dynamic metatheoretical framework, building on the theoretical work of Lewin, Vygotsky, Werner & Kaplan, we examined the evolving lifespace of the person, and their many subregions, as well as their variations. We proposed a new conceptual framework constituted by people’s domains of conduct, proximal and distal, and their degree of formalisation, ranging from informal personal domains into cultural and formal domains. Due to our access to unique longitudinal qualitative data, complemented with ethnographic observations, we were able to put this system to work and reveal two groups of developmental principles.

First, people develop and change when their relationship with the world is transformed. This can happen in three main ways: when the environment around them changes (such as during times of crisis), when they move to different environments, or when they experience personal ruptures triggered by events happening either inside themselves or in their external world.

Second, a variety of dynamics, that is, reconfigurations of the lifespace, can occur as a consequence. We have identified five dynamics: (1) domains of conduct can gain and loose importance, appear or disappear; (2) at a given moment in time, one or many can become more or less differentiated – some may even become more differentiated as others become less so. (3) As a consequence, when these are socially shared and culturally guided, personal domains can become cultural, or even formal domains – but these degrees of organisation can also be lost with dedifferentiation. (4) Domains of conducts may fuse, become subsumed to a larger one, or split. (5) Finally, proximal domains of conduct can become distal ones and vice versa.

This vocabulary can be applied to any life trajectory; it enables us to distinguish contrasting forms of courses of life, different modes of sense-making and uses of resources in the life of persons. Hence, although the two diarists we studied share many similarities – men in their seventies, single, engaged in their hobbies, with a past mobile life, having achieved a form of contentment - their way of handling their present is very different, one being very social and constantly imagining new futures, the other one more isolated and actively reminiscing.

Our proposed theoretical model enabled us to demonstrate that, when exposed to crises and ruptures, people may not only use a variety of resources to support their transitions, as has been widely shown (de Abreu et al., 2012; Gfeller & Grossen, 2025; Hale, 2008; Märtsin, 2019), but people also actively transform their material and symbolic environments, so as to reinforce the overall consistency of their lifespaces. We have shown how one diarist actively creates the material and social boundaries, or envelopes, of his lifespace, enabling him to maintain his agile psychological mobility in the past and future, while another diarist is using photography as a way to make the environment a symbolic and aesthetic place, which also has a stabilising function.

In more general terms, the present theoretical model accounts for developmental dynamics throughout the lifecourse. Although the emphasis was here on adult years, we have provided concepts that can account for developmental dynamics at any period in the course of life. Of course, in childhood and youth, we would probably observe the progressive emergence of new domains of conduct and their progressive differentiation, the slow formalisation of knowledge acquired at school, the tensions that may emerge within and between domains of conduct in adolescent years – all phenomena widely known under diverse theorisations. Our emphasis on the last third of the lifecourse in contrast reveals phenomena less familiar to developmental psychologists: that some domains of conducts may progressively loose importance, or be temporarily backgrounded; that new domains may emerge even in older age; that domains may fuse or subdivide, or become more or less distal; it shows that interpersonal differences may, with time, become stronger, even between people who superficially seem dealing with comparable circumstances. Our proposed model provides a general vocabulary that can account for the specificity of each person’s course of life, in their unique changing environments.

As corollary, this approach brings forward new observations. First, it confirms the fact that societal crises do not always produce the same personal ruptures. Second, it underlines the deep connection, that has only intuited so far, between geographical mobility and psychological change (Beckstead, 2010; Gillespie, 2006; Gillespie & Zittoun, 2015). In other words, moving through places and settings at times implies the emergence of new domains of conducts, or the reconfiguration of one’s lifespace. Third, it foregrounds a new phenomenon, so far only studied in specific cases (e.g., home making, dwelling) (Gfeller & Grossen, 2025; Märtsin, 2023): that people may engage in stabilising their material or material environment, with the use of material objects or semiotic elements as resources. People create external envelopes, or boundaries, for their lifespace to support their configuration of domains of conduct, especially in times of crises and ruptures.

Overall, the proposed theoretical framework expands and goes beyond the existing general theories of development in the lifecourse. For instance, the inspiring studies of life themes initiated by Charlotte Bühler did not account for the many different domains and their changes that may be invested diachronically and synchronically in a life (Bühler, 1968; Csikszentmihalyi & Beattie, 1979). The seminal study of Paul Baltes and his team, although it brought to the fore the importance of development to the end of life, ended up focusing more on biological decline and, through the SOC (selection, optimisation, compensation) model, proposed a normative model of development (Baltes, 1997; Baltes et al., 2006). In contrast, our approach shows that people may invest many domains, even without “optimising” them, replacing some with others, and keep developing some while others may indeed lose importance or require local compensation. Our model brings complexity by replacing a linear model with a multidimensional model, within an open dynamic approach. To theories underlying the narrative nature of the construction of the lifecourse, we bring a more situated, embodied, and dialogical perspective (Habermas, 2022; Martin, 2013). Also, because of the emphasis our theoretical frame has on dynamics, it goes beyond any attempts to reduce a person’s course of life to personality traits or attachment style (Henry et al., 2022; Magai & Haviland-Jones, 2002). Our approach keeps open the possibility that persons may, in different circumstances, develop ever changing modes of conduct and relating to themselves, others and the world. In addition, our approach proposes an integrative view of human conduct which goes beyond most approaches focusing either on cognitive development and/or affects (Carstensen, 2019; Thomas, 2020), or on sense-making and/or activity (Salvatore, 2019; Sato et al., 2013): it attempts to consider all these aspects at once. Finally, even to theories that emphasise the heterogeneity of people (Bamberg, 2011; Gergen, 1998; Habermas & de Silveira, 2008; Hermans, 2011), we propose a model where these diverse aspects are considered diachronically. All in all, we propose that people’s experience of the person are not united in one trajectory; rather, we claim that people’s domains can have very diverse and yet simultaneous trajectories. People are embedded in multiple domains of conducts and each domain can have its own trajectory, potentially interacting with other trajectories. Thus, it is a ‘bundle’, theory of development - which contrasts strongly with the highly bounded theories of development (e.g. as a singular trajectory). A possibly metaphor for such a developmental model (Leary, 1994; Zittoun & Gillespie, 2020) could be that of ivy: an organic entity, ivy is able to grow in very different circumstances and fed with various resources; it generates many branches that have they own trajectories yet that can cross each other and can grow together; they can adjust to many obstacles and changing circumstances, and yet some lines can get interrupted; all together, an ivy transforms the tree or the house on which it grows, and the overall landscape.

In summary, we have proposed a complex, multidimensional, dynamic, situated model of human development. What confers solidity to the present theorisation is its grounding in naturally occurring multi-decade qualitative data, constituted by online diaries, and complemented by ethnographic work. This has enabled us to combine diachronic and synchronic, width and breadth, as well as first and third person perspective, together with the multiresolution method of moving back-and-forth between macro patterns and micro events within the same data (Gillespie et al., 2024). We believe that such data, which is in addition based on publicly accessible material, provides great potential to the advancement of the study of human development over many decades.

## Data Availability

No datasets were generated or analysed during the current study.

## Footnotes

1 In other papers we have used the terms “spheres of experiences” to distinguish, initially, any domain of conduct (Zittoun, 2012, 2022 ; Zittoun & Gillespie, 2016); later we have restricted the term to subjective domains, distinguishing them from “cultural subsystem” and “formal knowledge” (Zittoun, 2022, 2023). The present proposition aims at bringing more clarity and consistency to the model. To further facilitate the reading, we sometimes use the term “domain” for “domain of conduct”.

## References

[CR1] Allport, G. W. (1942). *The use of personal documents in psychological science: Prepared for the committee on appraisal of research*. Social Science Research Council.

[CR2] Anzieu, D. (2007). Introduction à l’étude des enveloppes psychiques. *Psychanalyse des limites* (pp. 225–239). Dunod.

[CR3] Anzieu, D. (Ed.). (2003). *Les contenants de pensée*. Dunod.

[CR4] Baltes, P. B. (1997). On the incomplete architecture of human ontogeny. Selection, optimization and compensation of developmental psychology. *American Psychologist*, *52*(4), 266–380. 10.1037//0003-066x.52.4.366 9109347

[CR5] Baltes, P. B., Staudinger, U. M., & Lindenberger, U. (1999). Lifespan psychology: Theory and application to intellectual functioning. *Annual Review of Psychology*, *50*, 471–507. 15012462 10.1146/annurev.psych.50.1.471

[CR6] Baltes, P. B., Lindenberger, U., & Staudinger, U. M. (2006). Life span theory in developmental psychology. In W. Damon, & R. M. Lerner (Eds.), *Handbook of child psychology: Vol. 1. Theoretical models of human development* (6th ed., pp. 569–664). Wiley.

[CR7] Bamberg, M. (2011). Who am I? Narration and its contribution to self and identity. *Theory & Psychology*, *21*(1), 3–24. 10.1177/0959354309355852

[CR8] Beckstead, Z. (2010). Commentary: Liminality in acculturation and pilgrimage: When movement becomes meaningful. *Culture & Psychology*, *16*(3), 383–393. 10.1177/1354067X10371142

[CR9] Benson, C. (1993). *The absorbed self*. Harvester Wheatsheaf.

[CR10] Bernal Marcos, M. J., Zittoun, T., & Gillespie, A. (2024). Diaries as technologies for sense-making and self-transformation in times of vulnerability. *Integrative Psychological and Behavioral Science*, *58*(2), 563–588. 10.1007/s12124-023-09765-0 37014513 10.1007/s12124-023-09765-0 PMC10071254

[CR11] Bernal Marcos, M. J., Zittoun, T., & Gillespie, A. (2025). Beyond narratives of decline and success: Retirement through the lens of a diary. European Journal for Psychology of Education 40(3), 76. 10.1007/s10212-025-00973-3

[CR12] Bonnet, M., & Louchard Chardon, C. (Eds.). (2024). *Vieillissement et enveloppes psychiques*. In Press.

[CR13] Brown, S. D., & Reavey, P. (2020). Memory in the wild.: Lifes spaces, setting-specificity, and ecologies of experience. In B. Wagoner, de I. Brescó, & S. Zadeh (Eds.), *Memory in the wild* (pp. 3–56). Information Age Publishing.

[CR14] Bühler, C. (1968). The course of human life as a psychological problem. *Human Development*, *11*(3), 184–200. 5663537 10.1159/000270606

[CR15] Cangià, F. (2018). Precarity, imagination, and the mobile life of the ‘trailing spouse’. *Ethos*, *46*(1), 8–26. 10.1111/etho.12195

[CR16] Cangià, F. (2021). *Liminal moves: Traveling along places, meanings, and times*. Berghahn Books. https://www.berghahnbooks.com/title/CangiaLiminal

[CR17] Carstensen, L. L. (2019). Integrating cognitive and emotion paradigms to address the paradox of aging. *Cognition & Emotion*, *33*(1), 119–125. 10.1080/02699931.2018.1543181 30394173 10.1080/02699931.2018.1543181 PMC6476329

[CR18] Chang, E. S., Shane, J., Masdonati, J., & Villarreal, B. J. (2023). Editorial: Social relationships and career development throughout the lifespan: Identifying patterns of shared and non-shared agency. *Frontiers in Psychology*, *14*. 10.3389/fpsyg.2023.1235829 10.3389/fpsyg.2023.1235829 PMC10416231 37575419

[CR19] Cole, M. (2023). Prolepsis as a coordinating mechanism of semiotic mediation. *Constructivist Foundations*, *18*(2), Article2.

[CR20] Cornish, F. (2020). Towards a dialogical methodology for single case studies. *Culture & Psychology*, *26*(1), 139–152. 10.1177/1354067X19894925

[CR21] Cornish, F., Zittoun, T., & Gillespie, A. (2007). A cultural psychological reflection on collaborative research. Conference Essay: ESF Exploratory Workshop on Collaborative Case Studies for a European Cultural Psychology. *Forum Qualitative Sozialforschung/Forum: Qualitative Social Research*, *8*(3), Art 21. [37 paragraphs].

[CR22] Cornish, F., Gillespie, A., & Zittoun, T. (2013). Collaborative analysis of qualitative data. In U. Flick (Ed.), *The SAGE handbook of qualitative data analysis* (pp. 79–93). Sage.

[CR23] Crafter, S., Maunder, R., & Soulsby, L. (2019). *Developmental transitions: Exploring stability and change through the lifespan*. Routledge. 10.4324/9781315625263

[CR24] Csikszentmihalyi, M., & Beattie, O. V. (1979). Life-themes: A theoretical and empirical exploration of their origins and effects. In M. Csikszentmihalyi (Ed.), *Applications of flow in human development and education. The collected works of Mihaly Csikszentmihalyi* (Original 1979, pp. 85–97). Springer.

[CR25] de Abreu, G., O’Sullivan-Lago, R., & Hale, H. C. (2012). Nowadays i think, Wow: I made it’: Exporing immigrant transitions drawing on dialogical self theory and the notion of symbolic resources. In M. César & B. Ligorio (Eds.), *The interplays between dialogical learning and dialogical self* (pp. 127–150). InfoAge.

[CR26] Dewey, J. (1946). Interaction and transaction. *The Journal of Philosophy*, *43*(19), 505–517.

[CR27] Di Donato, F., Lavanchy, A., Garros, E., Mahon, P., & Zittoun, T. (2020). *La fabrique de l’intégration*. Antipodes. https://www.antipodes.ch/telechargement_pdf/didonato-garros-lavanchy-mahon-zittoun_la-fabrique-de-l-integration_978-2-88901-974-8_10.33056-ANTIPODES.11704.pdf

[CR28] Erikson, E. H. (1959). *Identity and the life cycle. Selected papers.* (1959th edn). International Universities Press. http://www.archive.org/details/identityandtheli011578mbp

[CR29] Flyvbjerg, B. (2006). Five misunderstanding about case-study research. *Qualitative Inquiry*, *12*(2), 219–245.

[CR30] Fuller-Iglesias, H. R., Webster, N. J., & Antonucci, T. C. (2015). The complex nature of family support across the life span: Implications for psychological well-being. *Developmental Psychology*, *51*(3), 277–288. 10.1037/a0038665 25602936 10.1037/a0038665 PMC4497824

[CR31] Geertz, C. (1972). La religion comme système culturel. In R. E. Bradbury, C. Geertz, M. E. Spiro, V. W. Turner, & E. H. Winter (Eds.), *Essais d’anthropologie religieuse* (pp. 19–66). Gallimard.

[CR32] Gergen, J. K. (Ed.). (1998). *Toward the relational self*. American Psychological Association.

[CR33] Gfeller, F., & Grossen, M. (2025). Moving house in older age: Challenges and resources for the sense of self-continuity. In T. Zittoun, & F. Gfeller (Eds.), *Ageing, learning and Development. Sociocultural and psychoanalytical perspectives on growing older*. Springer.

[CR34] Gillespie, A. (2006). *Becoming other: From social interaction to self-reflection*. Information Age Publishing.

[CR35] Gillespie, A., & Zittoun, T. (2010). Studying the movement of thought. In A. Toomela, & J. Valsiner (Eds.), *Methodological thinking in psychology: 60 years gone astray?* (pp. 69–88). Information Age.

[CR36] Gillespie, A., & Zittoun, T. (2015). Social and psychological movement: Weaving individual experience into society. In B. Wagoner, N. Chaudhary, & P. Hviid (Eds.), *Integrating experiences: Body and Mind moving between contexts* (pp. 279–294). Information Age Publishing.

[CR37] Gillespie, A., Glăveanu, V., de Saint-Laurent, C., Zittoun, T., & Bernal Marcos, M. J. (2024). Multi-resolution design: Using qualitative and quantitative analyses to recursively zoom in and out of the same dataset. *Journal of Mixed Methods Research*, 15586898241284696. 10.1177/15586898241284696

[CR38] Goffman, E. (1974). *Frame Analysis: An Essay on the Organization of Experience* (New edition). Harper & Row.

[CR39] Greco Morasso, S., & Zittoun, T. (2014). The trajectory of food as a symbolic resource for international migrants. *Outlines Critical Practice Studies*, *15*(1), 28–48.

[CR40] Grossen, M. (2015). The diary as dialogical space. In B. Wagoner, N. Chaudhary, & P. Hviid (Eds.), *Integrating experience. Body and Mind moving between contexts* (pp. 201–219). Information Age Publishing.

[CR41] Habermas, T. (2022). *The longitudinal study of brief life narratives: MainLife Study (2002–2019) Study Report. Qualiservice* & *GESIS.*10.26092/elib/1651

[CR42] Habermas, T., & de Silveira, C. (2008). The development of global coherence in life narratives across adolescence: Temporal, causal, and thematic aspects. *Developmental Psychology*, *44*(3), 707–721. 10.1037/0012-1649.44.3.707 18473638 10.1037/0012-1649.44.3.707

[CR43] Hale, H. C. (2008). The development of British military masculinities through symbolic resources. *Culture & Psychology*, *14*(3), 305–332. 10.1177/1354067X08092636

[CR44] Henry, A., Allain, P., & Potard, C. (2022). Relationships between theory of Mind and attachment styles in emerging adulthood. *Journal of Adult Development*, *29*(3), 179–191. 10.1007/s10804-022-09399-3

[CR45] Hermanowicz, J. C. (2016). Longitudinal qualitative research. In M. J. Shanahan, J. T. Mortimer, & M. Kirkpatrick Johnson (Eds.), *Handbook of the Life Course: Volume II* (pp. 491–513). Springer International Publishing. 10.1007/978-3-319-20880-0_22

[CR46] Hermans, H. J. M. (2011). The dialogical self. In S. Gallagher (Ed.), *The Oxford handbook of the self*. Oxford University Press. http://www.oxfordhandbooks.com/view/10.1093/oxfordhb/9780199548019.001.0001/oxfordhb-9780199548019-e-29

[CR47] Hviid, P. (2022). Aged experience – A cultural developmental investigation. *Learning Culture and Social Interaction*, *37*, 100386. 10.1016/j.lcsi.2020.100386

[CR48] Janet, P. (2003). *De l’angoisse* à *l’extase. Étude sur les croyances et les sentiments: Vol. II* (Original edition 1927). Librairie Félix Alcan. http://classiques.uqac.ca/classiques/janet_pierre/angoisse_extase_2/angoisse_2.html

[CR49] Kaës, R. (2007). Du Moi-peau aux enveloppes psychiques. Genèse et développement d’un concept. *Le Carnet Psy*, *117*(4), 33–39. 10.3917/lcp.117.0033

[CR50] Leary, D. E. (1994). *Metaphors in the history of psychology*. Cambridge University Press.

[CR51] Lejeune, P. (1975). *Le pacte autobiographique*. Le Seuil.

[CR52] Lejeune, P. (2000). *‘Cher écran⋯’ Journal personnel, ordinateur, internet*. Seuil.

[CR53] Lemogne, C., Piolino, P., Jouvent, R., Allilaire, J. F., & Fossati, P. (2006). Mémoire autobiographique épisodique et dépression: Episodic autobiographical memory in depression: A review. *L’Encéphale*, *32*(5, Part 1), 781–788. 10.1016/S0013-7006(06)76231-5 17099603 10.1016/s0013-7006(06)76231-5

[CR54] Lewin, K. (1936). *Principles of topological psychology* (F. Heider & G. M. Heider, Trans.). McGraw-Hill Book Company, Inc.

[CR55] Lewin, K. (1940). Formalization and progress in psychology. In *Resolving social conflicts* & *Field theory in social science* (Original publication 1940, pp. 168–190). American Psychological Association (APA).

[CR56] Lewin, K. (1951). Behavior and development as function of the total situation. In E. Cartwright (Ed.), *Field theory in the social sciences* (Original 1946, pp. 238–303). Harper and brothers publishers.

[CR57] Lightfoot, C., Lalonde, C., & Chandler, M. (Eds.). (2004). *Changing conceptions of psychological life*. Lawrence Erlbaum Associates. 10.4324/9781410610966

[CR58] Magai, C., & Haviland-Jones, J. (2002). *The hidden genius of emotion: Lifespan transformations of personality*. Cambridge University Press.

[CR59] Marková, I. (2016). *The dialogical mind: Common sense and ethics*. Cambridge University Press.

[CR60] Martin, J. (2013). Life positioning analysis: An analytic framework for the study of lives and life narratives. *Journal of Theoretical and Philosophical Psychology*, *33*(1), 1–17. 10.1037/a0025916

[CR61] Märtsin, M. (2019). Identity development in the lifecourse: A semiotic cultural approach to transitions in early adulthood. *Palgrave Macmillan*. 10.1007/978-3-030-27753-6

[CR62] Märtsin, M. (2023). Homemaking away from home: A semiotic cultural psychology perspective. *Frontiers in Psychology*, *14*, 1215654–1215654. 10.3389/fpsyg.2023.1215654 38144976 10.3389/fpsyg.2023.1215654 PMC10739469

[CR63] Montero, B. G. (2016). *Thought in action: Expertise and the conscious Mind*. Oxford University Press. 10.1093/acprof:oso/9780199596775.001.0001

[CR64] Muller Mirza, N. (2026 ). Developmental processes and meaning-making in volunteering activity. In T. Zittoun, & F. Gfeller (Eds.), *Ageing, learning and Development. Sociocultural and psychoanalytical perspectives on growing older*. Springer.

[CR65] Nell, V. (1988). *Lost in a book: The psychology of reading for pleasure*. Yale University Press.

[CR66] Pedersen, O. C. (2025). Maintaining the future through recurrent crises. *Culture & Psychology*, *31*(1), 96–115. 10.1177/1354067X231204298 40040690 10.1177/1354067X231204298 PMC11872673

[CR67] Pedersen, O. C., & Zittoun, T. (2022). I have been born, Raised and lived my whole life here – perpetually on the move while remaining still. *Integrative Psychological and Behavioral Science*, *56*(3), 755–778. 10.1007/s12124-021-09660-6 34782992 10.1007/s12124-021-09660-6 PMC8592804

[CR68] Pedersen, O. C., Perrin, M., & Zittoun, T. (submitted paper). *Resonating crises: A longitudinal study of ruptures in times of crises*.

[CR69] Piaget, J. (1977). *The development of thought: Equilibration of cognitive structures*. Viking.

[CR70] Riessman, C. K. (1993). *Narrative analysis* (1st ed.). Sage Publications, Inc.

[CR71] Rosenthal, G. (1993). Reconstruction of life stories: Principles of selection in generating stories for narrative biographical interviews. *The Narrative Study of Lives*, *1*(1), 59–91.

[CR72] Salvatore, S. (2019). Beyond the meaning Given. The meaning as explanandum. *Integrative Psychological and Behavioral Science*. 10.1007/s12124-019-9472-z 30712225 10.1007/s12124-019-9472-z

[CR73] Sato, T., Yasuda, Y., Kanzaki, M., & Valsiner, J. (2013). From describing to reconstructing life trajectories: How the TEA (Trajectory equifinality Approach) explicates context-dependent human phenomena. In B. Wagoner, N. Chaudhary, & P. Hviid (Eds.), *Cultural psychology and its future: Complementarity in a new key* (pp. 93–105). Information Age Publishing.

[CR74] Schubauer-Leoni, M. L., Grossen, M., & Perret-Clermont, A. N. (1992). The construction of adult child intersubjectivity in psychological research and in school. In von M. Cranach, W. Doise, & G. Mugny (Eds.), *Social representations and the social bases of knowledge* (pp. 69–77). Hogrefe & Huber. http://doc.rero.ch/record/21312?ln=fr

[CR75] Schuetz, A. (1944). The stranger: An essay in social psychology. *American Journal of Sociology*, *49*(6), 499–507. 10.2307/2771547

[CR76] Schuetz, A. (1945). The homecomer. *American Journal of Sociology*, *50*(5), 369–376. 10.2307/2771190

[CR77] Schütz, A., & Luckmann, T. (1973). *The structures of the life-world*. Northwestern University.

[CR78] Stern, D. N. (2004). *The present moment in psychotherapy and everyday life*. W. W. Norton & Company.

[CR79] Svačinová, I. (2021). Pragma-dialectical reconstruction of crisis diary-writing as a communicative activity type. *Argumentation*, *35*(2), 237–264. 10.1007/s10503-020-09524-0

[CR80] Theisen-Womersley, G. (2021). Trauma and resilience among displaced populations: A Sociocultural exploration. *Springer International Publishing*. 10.1007/978-3-030-67712-1

[CR81] Thomas, A. K. (Ed.). (2020). *The Cambridge handbook of cognitive aging: A life course perspective*. Cambridge University Press.

[CR82] Trevarthen, C. (2012). Embodied human intersubjectivity: Imaginative agency, to share meaning. *Cognitive Semiotics*, *4*(1), 6–56.

[CR83] Valsiner, J. (1998). *The guided mind: A sociogenetic approach to personality*. Harvard University Press.

[CR84] Valsiner, J. (2021). *General human psychology*. Springer Nature Switzerland AG.

[CR85] Valsiner, J. (2024). Dynamic semiosis. *Springer Nature Switzerland*. 10.1007/978-3-031-75602-3

[CR86] Valsiner, J., Molenaar, P. C. M., Lyra, M. C. D. P., & Chaudhary, N. (2009). *Dynamic process methodology in the social and developmental sciences* (1st ed.). Springer.

[CR87] Van Geert, P. (2009). Nonlinear complex dynamical systems in developmental psychology. In S. J. Guastello, M. Koopmans, & D. Pincus (Eds.), *Chaos and complexity in psychology. The theory of nonlinear dynamical systems* (pp. 242–271). Cambridge University Press.

[CR88] van Geert, P. L. C. (2019). Dynamic systems, process and development. *Human Development*, *63*(3–4), 153–179. 10.1159/000503825 32139922 10.1159/000503825 PMC7050700

[CR89] Van Vreeswijk, M. F., & De Wilde, E. J. (2004). Autobiographical memory specificity, psychopathology, depressed mood and the use of the autobiographical memory test: A meta-analysis. *Behaviour Research and Therapy*, *42*(6), 731–743. 10.1016/S0005-7967(03)00194-3 15081887 10.1016/S0005-7967(03)00194-3

[CR90] Vygotsky, L. S. (1934). In E. Hanfmann, G. Vakar, & N. Minnick (Eds.), *Thinking and speech*. Trans.). MIT Press. https://www.marxists.org/archive/vygotsky/works/words/index.htm

[CR91] Werner, H., & Kaplan, B. (1963). *Symbol formation. An organismic-developmental approach to Language and the expression of thought*. John Wiley & son.

[CR92] Winnicott, D. W. (1991). *Playing and reality* (Original 1971). Routledge.

[CR93] Winnicott, D. W. (1994). *Holding and interpretation: Fragment of an analysis*. Grove.

[CR94] Witherington, D. C. (2007). The dynamic systems approach as metatheory for developmental psychology. *Human Development*, *50*(2–3), 127–153.

[CR95] Witherington, D. C. (2015). Dynamic systems in developmental science. *Handbook of child psychology and developmental science* (pp. 1–50). John Wiley & Sons, Ltd. 10.1002/9781118963418.childpsy103

[CR96] Witherington, D. C., & Boom, J. (2019). Conceptualizing the dynamics of development in the 21st century: Process, (inter)action, and complexity. *Human Development*, *63*(3–4), 147–152. 10.1159/000504097

[CR97] Zittoun, T. (2006). *Transitions. Development through symbolic resources*. Information Age Publishing.

[CR98] Zittoun, T. (2008). Janet’s emotions in the whole of human conduct. In R. Diriwaechter, & J. Valsiner (Eds.), *Striving for the whole: Creating theoretical synthesis* (pp. 111–129). Transaction.

[CR99] Zittoun, T. (2009). Dynamics of life-course transitions – a methodological reflection. In J. Valsiner, P. C. M. Molenaar, M. C. D. P. Lyra, & N. Chaudhary (Eds.), *Dynamic process methodology in the social and developmental sciences* (pp. 405–430). Springer.

[CR100] Zittoun, T. (2012). Lifecourse. In J. Valsiner (Ed.), *Handbook of culture and psychology* (pp. 513–535). Oxford University Press.

[CR101] Zittoun, T. (2014). Three dimensions of dialogical movement. *New Ideas in Psychology*, *32*, 99–106. 10.1016/j.newideapsych.2013.05.006

[CR102] Zittoun, T. (2019). A Sociocultural psychological approach to religion. *Integrative Psychological and Behavioral Science*, *53*(1), 107–125. 10.1007/s12124-018-9457-3 30178227 10.1007/s12124-018-9457-3

[CR103] Zittoun, T. (2020). Imagination in people and societies on the move: A Sociocultural psychology perspective. *Culture & Psychology*, *26*(4), 654–675. 10.1177/1354067X19899062

[CR104] Zittoun, T. (2022). A Sociocultural psychology of the life course to study human development. *Human Development*, *66*(4–5), 306–324. 10.1159/000526435

[CR105] Zittoun, T. (2023). *The pleasure of thinking*. Cambridge University Press. 10.1017/9781009039802

[CR106] Zittoun, T. (2026a). On being an oxymoron: A Sociocultural psychology of development with older age. In T. Zittoun, & F. Gfeller (Eds.), *Ageing, learning and Development. Sociocultural and psychoanalytical perspectives on growing older*. Springer.

[CR107] Zittoun, T. (2026b). *Developing in older age* – *an integrative view*. Cambridge University Press.

[CR108] Zittoun, T., & Gillespie, A. (2012). Using diaries and self-writings as data in psychological research. In E. Abbey, & S. E. Surgan (Eds.), *Emerging methods in psychology* (pp. 1–26). Transaction.

[CR109] Zittoun, T., & Gillespie, A. (2015a). Integrating experiences: Body and Mind moving between contexts. In B. Wagoner, N. Chaudhary, & P. Hviid (Eds.), *Integrating experiences: Body and Mind moving between contexts* (pp. 3–49). Information Age Publishing.

[CR110] Zittoun, T., & Gillespie, A. (2015b). Transitions in the lifecourse: Learning from Alfred Schütz. In A. C. Joerchel, & G. Benetka (Eds.), *Biographical ruptures and their repairs: Cultural transitions in development* (pp. 147–157). Information Age.

[CR111] Zittoun, T., & Gillespie, A. (2016). *Imagination in human and cultural development*. Routledge.

[CR112] Zittoun, T., & Gillespie, A. (2020). Metaphors of development and the development of metaphors. *Theory & Psychology*, *30*(6), 827–841. 10.1177/0959354320939194

[CR113] Zittoun, T., & Gillespie, A. (2022). A Sociocultural approach to identity through diary studies. In M. Bamberg, C. Demuth, & M. Watzlawik (Eds.), *Cambridge handbook of identity* (pp. 345–365). Cambridge University Press.

[CR114] Zittoun, T., Baucal, A., Cornish, F., & Gillespie, A. (2007). Collaborative research, knowledge and emergence. *Integrative Journal for Psychological and Behavioral Science*, *41*(2), 208–217. 10.1007/s12124-007-9021-z 18193522

[CR115] Zittoun, T., Valsiner, J., Vedeler, D., Salgado, J., Gonçalves, M., & Ferring, D. (2013). *Human development in the lifecourse. Melodies of living*. Cambridge University Press.

[CR116] Zittoun, T., Grossen, M., & Tarrago Salamin, F. (2021). Creating new spheres of experience in the transition to a nursing home. *Learning Culture and Social Interaction*, *28*, 100458. 10.1016/j.lcsi.2020.100458

[CR117] Zittoun, T., Gillespie, A., & Bernal Marcos, M. J. (2024). Development and vulnerability across the lifecourse. *Culture & Psychology*, *30*(4), 813–840. 10.1177/1354067X231201384 39502649 10.1177/1354067X231201384 PMC11532022

